# Investigation of the protective mechanism of leonurine against acute myocardial ischemia by an integrated metabolomics and network pharmacology strategy

**DOI:** 10.3389/fcvm.2022.969553

**Published:** 2022-08-22

**Authors:** Weiwei Rong, Jiejia Li, Lifeng Wang, Shanshan Luo, Tulu Liang, Xunjia Qian, Xiaodan Zhang, Qinbei Zhou, Yizhun Zhu, Qing Zhu

**Affiliations:** ^1^School of Pharmacy, Nantong University, Nantong, China; ^2^Provincial Key Laboratory of Inflammation and Molecular Drug Target, Nantong, China; ^3^School of Pharmacy and State Key Laboratory for the Quality Research of Chinese Medicine, Macau University of Science and Technology, Macau, Macau SAR, China; ^4^Shanghai Key Laboratory of Bioactive Small Molecules, Department of Pharmacology, School of Pharmacy, Fudan University, Shanghai, China; ^5^Research Center for Intelligent Information Technology, Nantong University, Nantong, China

**Keywords:** leonurine, network pharmacology, metabolomics, acute myocardial ischemia, molecular docking

## Abstract

**Background:**

Leonurus japonicus Houtt has an obvious efficacy on cardiovascular diseases. As the most representative component in the herb, leonurine has attracted increasing attention for its potential in myocardial ischemia. However, its protective mechanism against myocardial ischemia remains incompletely elucidated.

**Objectives:**

The present study aimed to reveal the potential mechanism of leonurine in acute myocardial ischemia using a strategy combining metabolomics and network pharmacology.

**Methods:**

First, a metabolomics method was proposed to identify the differential metabolites of plasma in rats. Then, network pharmacology was performed to screen candidate targets of leonurine against acute myocardial ischemia. A compound-reaction-enzyme-gene network was thus constructed with the differential metabolites and targets. Finally, molecular docking was carried out to predict the binding capability of leonurine with key targets.

**Results:**

A total of 32 differential metabolites were identified in rat plasma, and 16 hub genes were detected through network pharmacology. According to the results of compound-reaction-enzyme-gene network and molecular docking, what was screened included six key targets (GSR, CYP2C9, BCHE, GSTP1, TGM2, and PLA2G2A) and seven differential metabolites (glycerylphosphorylcholine, lysophosphatidylcholine, choline phosphate, linoleic acid, 13-HpODE, tryptophan and glutamate) with four important metabolic pathways involved: glycerophospholopid metabolism, linoleic acid metabolism, tryptophan metabolism and glutamate metabolism. Among them, glycerophospholipid and tryptophan metabolism were shown to be important, since the regulation of leonurine on these two pathways was also observed in our previous metabolomics study conducted on clinical hyperlipidemia patients.

**Conclusion:**

This is the first study of its kind to reveal the underlying mechanism of leonurine against acute myocardial ischemia through a strategy combining metabolomics and network pharmacology, which provides a valuable reference for the research on its future application.

## Graphical Abstract

The strategy to reveal the potential mechanism of leonurine against AMI by combining with metabolomics and network pharmacology. 
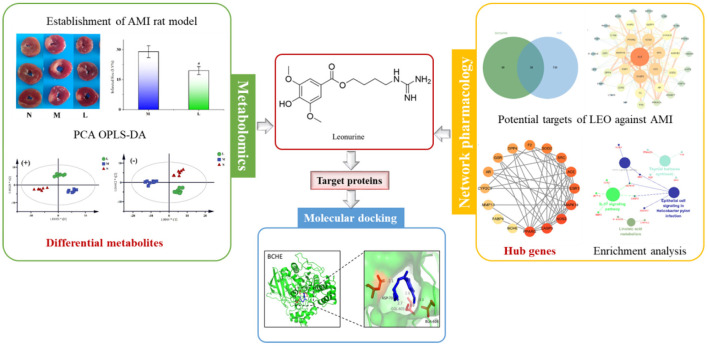

## Highlights

- A compound-reaction-enzyme-gene network was constructed.
- Not only differential metabolites affected by leonurine were found, the upstream targets and pathways were also predicted.
- Some metabolic pathways of this experiment can be verified with previous clinical metabolomics study of leonurine.

## Introduction

Coronary atherosclerotic heart disease (CHD), commonly known as ischemic heart disease, is the myocardial damage caused by the change in cardiac coronary circulation, which can result in an imbalance between coronary blood flow and myocardial demand. Ischemic heart disease, especially acute myocardial infarction (AMI), is a serious disease threatening human health due to its severe harmfulness to cardiac tissues and high mortality (1). As a commonly seen disease, CHD has a high morbidity and mortality worldwide. To compound it, stress, unhealthy diet and lifestyle cause CHD to occur in a growing number of young people in many countries, which poses a serve threat to human health (2, 3). Therefore, it is imperative to increase effort on the research and development of anti-myocardial ischemic drugs worldwide.

During ischemia myocardial, reactive oxygen species (ROS) with high reactivity and toxicity are produced, which can exacerbate the degree of myocardial damage when myocardial ischemia occurs (4–6). Leonurus japonicus Houtt. (also known as Herba Leonuri or motherwort) has been used for thousands of years in China. As a classic herbal medicine, motherwort has a positive effect in the treatment of a variety of diseases, including cardiovascular diseases. A modern medical study found that the excellent antioxidant activity of motherwort extract can reduce the damage caused by ROS and exert cardioprotection during myocardial ischemia (5). In motherwort, alkaloids are considered the most crucial bioactive components, especially leonurine (also known as SCM-198 in our group), which is the main contributor to the antioxidant activity of motherwort extract (7–11). Due to its importance, leonurine was even recorded in the Chinese pharmacopeia as an official index for measuring the quality of motherwort (12). Apart from its significant antioxidant activity, leonurine also exerts cardioprotective effects via other mechanisms. For example, leonurine attenuated apoptosis in cardiac muscle cells induced by hypoxia (13, 14), doxorubicin (15) or hydrogen peroxide (16). The anti-apoptotic effects of leonurine were probably mediated by activating the PI3K/Akt signaling pathway (17). In addition, leonurine prevented cardiac fibrosis in postmyocardial infarction rats through modulation of a Nox4-ROS pathway (10). What is even more encouraging is our discovery that the long-term use of leonurine could help improve the lipid profiles of multiple animal models including mouse, rabbit and rhesus monkeys (18), for which it is widely regarded as an indicator of coronary atherosclerotic heart disease risk, particularly low-density lipoprotein and cholesterol levels (19). As a promising novel drug, leonurine has now been applied in clinical trials in China for treating hyperlipidemia. Despite some achievements made in existing studies on leonurine in ischemic heart diseases, the protective mechanism and targets of leonurine against AMI remain incompletely understood.

It is well-known that cardiac metabolic alterations are the first consequences of AMI (20). For example, Bonanad et al. (21) identified up to 32 serum differential metabolites from swine (*n* = 9) and patients (*n* = 20) during AMI. Among them, creatine increased 2 h after ischemia. Untargeted metabolomics serves as an effective tool for the overall, systematic study of changes in metabolites *in vivo*. According to the statistical analysis of differential metabolites, metabolomics can reveal changes to endogenous metabolites in different physiological states or different physiological states before and after administration, which is essential for inferring the possible etiology or treatment mechanism. However, it is inadequate to infer the protective mechanism of leonurine against AMI based only on differential metabolites. Therefore, it is also necessary to reveal the upstream proteins causing the changes in metabolites and the possible targets of leonurine. Network pharmacology is applicable to systematically predict the possible targets of drugs with the support of statistics, complex networks and other mathematical methods. Although network pharmacology can be efficiently accounted for the mechanism of drugs on diseases from the perspective of systems biology, an independent network pharmacology experiment is limited to predicting the targets and pathways in mathematical dimensions, and there is a lack of statistical support from practical experiments.

**Figure 1:**
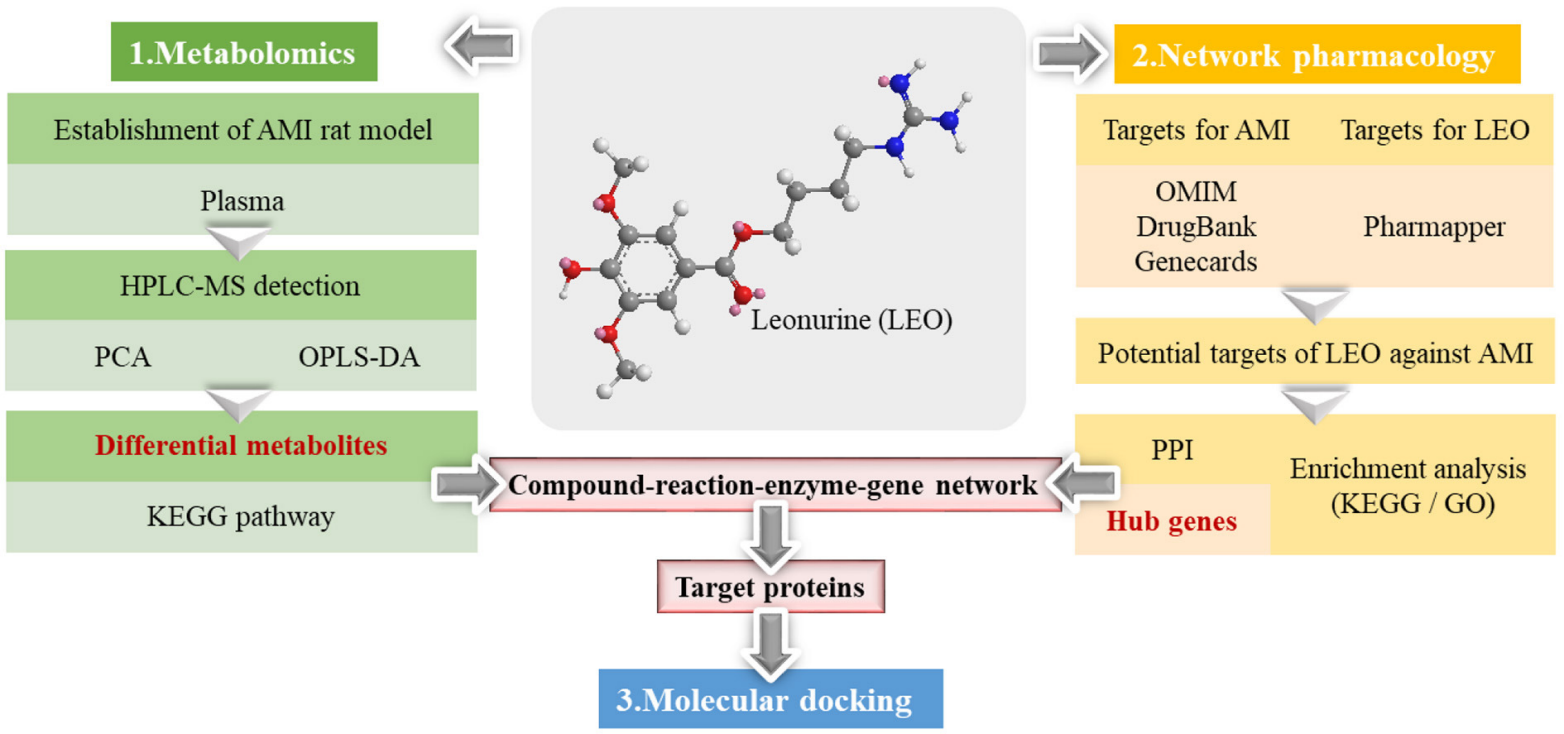
The workflow of the metabolomics and network pharmacological integration strategy for the study of the leonurine protective mechanism on AMI. The structure of leonurine is shown in the center position. The differential metabolites were first filtered by metabolomics; then, the hub genes were screened by network pharmacology; finally, the key targets were verified by molecular docking.

For this reason, a strategy combining metabolomics and network pharmacology was applied in this study to integrate the differential metabolites with the key targets effectively, thus establishing a compound-reaction-enzyme-gene network. This strategy was adopted not only to investigate the metabolite changes occurring after leonurine intervention, but also to predict the upstream potential targets that might make a difference to the metabolite levels. Additionally, the potential of key targets to bind with leonurine was also predicted using molecular docking technology. Finally, success was achieved in revealing the protective mechanism of leonurine on AMI. The workflow of the strategy is shown in Figure 1.

## Materials and methods

### Materials and reagents

Leonurine was synthesized by Professor Zhu Yizhun's research group of Fudan University School of Pharmacy, and its purity was verified as 98% by HPLC. The powder was prepared with normal saline at a concentration of 1.5 mg/mL and administered intraperitoneally at a dose of 15 mg/kg. The structure of leonurine is shown in Figure 1.

HPLC-grade acetonitrile, methanol and formic acid were provided by Merck, USA. Analytical grade chemical reagents were purchased from Shanghai Sinopharm Group Chemical Reagent Co., Ltd. Distilled water was supplied by Wahaha Co. Ltd. Creatine kinase-muscle/brain (CK-MB) and troponin I (Tn-I) kits were purchased from Nanjing Jiancheng Biotechnology Co., LTD.

### Animals and modeling

Twenty-four male Sprague–Dawley rats (200 ± 10 g) were supplied by the Experimental Animal Center of Nantong University. They were raised with food and water available *ad libitum* in an air-conditioned room with a temperature of 22 ± 2°C, a humidity of 55 ± 10% and a 12 h light-dark cycle for at least 7 days to adapt to the environment. Then the rats were randomly divided into three groups with eight rats in each group: the sham-operated group (N), the AMI model group (M) and the leonurine-treated group (L, *i.p*., 15 mg/kg/day). The results of our previous study showed leonurine with a dosage of 15 mg/kg/d could show significant antioxidant activity and exert cardioprotection during AMI (9, 10, 14). The drug solution was administered to rats in the L group preoperatively for 7 days before coronary ligation and immediately after coronary ligation on the same day. The rats in the N and M groups were given an intraperitoneal injection at the same volume of normal saline.

The rats were anesthetized by isoflurane and fixed in the supine position, and electrocardiogram (ECG) monitoring was performed during the surgery. After endotracheal intubation, artificial ventilation was performed. The intercostal muscle of the rats was separated, and a third intercostal thoracotomy was performed along the left margin of the sternum at 0.5 cm. The intercostal incision was opened with a chest dilator, and the pericardium was cut to expose the heart. The pleura was kept intact to preserve autonomous breathing. The left anterior coronary artery was permanently tied between the left atrial ear and the pulmonary artery cone at approximately 2.3 mm from the root of the aorta with a filament needle. If both myocardial whitening in the blood supply area and ST elevation of the standard limb guide ECG were observed, the model was successfully established (22). The chest was then quickly closed and autonomous breathing was resumed. The N group underwent the same operation except the coronary artery was not ligated. All studies on animals were in accordance with the Guidelines of the Committee on the Care and Use of Laboratory Animals in China.

### Sample collection and preparation

Heart and blood samples were collected 48 h after coronary ligation surgery. The blood samples were immediately centrifuged at 1,000×g for 15 min to obtain the supernatant, which was separated and stored at −80°C until analysis.

Plasma samples were thawed at room temperature before analysis. An aliquot of 300 μL of methanol was added to 100 μL of plasma and vortexed for ~3 min for protein precipitation. The mixture was centrifuged at 10,000×g for 15 min at 4°C. The supernatant was separated for LC–MS analysis. Quality control (QC) samples were prepared by mixing equal aliquots of each sample. One QC sample was injected every six samples.

Other plasma samples were taken and measured according to the instructions provided by CK-MB and Tn-I kits. The activity of CK-MB and the content of Tn-I were obtained with a microplate reader.

### Histological analysis

The ventricles of rats were cut into multiple horizontal slices with 2 mm thickness and heated in a water bath at 37°C in 1% 2,3,5-triphenyltetrazole chloride (TTC) solution for 10 min. The reaction was terminated with normal saline and then fixed in 4% paraformaldehyde solution for 30 min. Areas stained red indicated normal myocardium, while areas not stained red and that were pale were infarcted. The infarct and ventricular areas were calculated automatically by ImageJ software (Version 3.0) according to the different colors. All data are expressed as the mean ± SD. The results were analyzed by Student's *t*-test, and a *p*-value < 0.05 was considered to indicate statistical significance. Infarcted Size (LV%) = Infarct Area/Ventricular Area × 100

### Instruments, parameters, and conditions of metabolomics

An LC-Q/TOF-MS (Agilent, 1290 Infinity LC, 6530 UHD and Accurate-Mass Q-TOF/MS) system was used for sample detection. The separation was performed on a C18 chromatographic column (2.1 × 100 mm, 1.8 μm; Agilent, USA) at 40°C. The flow rate was set as 0.4 mL min^−1^, and the sample injection volume was 5 μL. The mobile phase system was composed of an A phase (0.1% formic acid) and a B phase (0.1% formic acid in acetonitrile). The gradient elution program started with 5% B for 2 min, increased from 5% B to 95% B for 12 min, and remained at 95% B for 2 min.

The raw data were collected in positive and negative electrospray ionization modes (ESI+/–). The source temperature in both modes was 100°C. In positive mode, the capillary voltage was set as 4,000 V, and the sampling cone was set as 3,500 V. The desolvation temperature was 350°C, and the extraction cone was 4 V. The cone and desolvation gas flow were 50 L/h and 600 L/h, respectively. In negative mode, the capillary voltage was set as 3,500 V, and the sampling cone was set as 5,000 V. The desolvation temperature was 300°C, and the desolvation gas flow was 700 L/h. The cone gas flow and extraction cone values were the same as those in positive mode. The scan time was 0.03 s, and interscan time was 0.02 s. The MS scans were ranged from 100 to 1,000 Da. To ensure the accuracy and repeatability of the mass, leucine-enkephalin was used as the lock mass. The [M+H]^+^ ion 556.2771 Da was produced in positive ion mode, and the [M–H]^−^ ion 554.2615 Da was produced in negative ion mode.

### Statistical analysis of metabolomics

The raw data were processed by MassHunter software. Filtration and peak identification, peak match across samples, and retention time correction were processed by XCMS code. The zero values were reduced based on the “80% rule” (23). Unsupervised principal component analysis (PCA) and supervised orthogonal partial least squares discrimination analysis (OPLS-DA) were used to screen the potential biomarkers by SIMCA software. Hierarchical cluster analysis (HCA) and metabolic pathway analysis were conducted by MetaboAnalyst 5.0 software and KEGG (http://www.genome.jp/kegg/), respectively. Student's *t*-test was used to perform statistical analysis, and the metabolites with variable influence on project (VIP) values >1 were considered to be differential metabolites. The HMDB database (http://www.hmdb.ca/) was applied to annotate the potential biomarkers via their mass information.

### Network pharmacology research

The experimental network pharmacology procedure is shown in Figure 1. In detail, the potential target search for leonurine was achieved by the PharmMapper Server (http://www.lilab-ecust.cn/pharmmapper/) (24). The related targets and candidate genes of AMI were obtained from the Drug Bank (https://go.drugbank.com) (25), OMIM (https://omim.org/) (26) and Genecards (https://www.genecards.org/) (27) websites using the keywords “myocardial ischemia” or “acute myocardial ischemia.” The intersection of candidate targets for AMI and leonurine was obtained, which was further inputted into the STRING website (https://string-db.org/) (28) to construct a protein–protein interaction (PPI) network (medium confidence > 0.4). Cytoscape software was also used for PPI network visualization. Hub genes were obtained using the CytoHubba plugin in Cytoscape software. Gene ontology (GO) enrichment and KEGG pathway (*p*-value < 0.05) analysis of potential targets were achieved by the ClueGO plugin. The compound-reaction-enzyme-gene network was established by Metscape plugin to visualize the interactions between metabolites, pathways, enzymes and genes, thereby identifying key metabolites and targets.

### Molecular docking

The 3D structure of leonurine was drawn with Chem3D software. The crystal structures of key protein targets were acquired from the RCSB Protein Data Bank website (https://www.rcsb.org/). A total of six protein targets were studied: cholinesterase (BCHE), protein-glutamine gamma-glutamyltransferase 2 (TGM2), glutathione S-transferase P (GSTP1**)**, cytochrome P450 2C9 (CYP2C9), phospholipase A2 (PLA2G2A) and glutathione reductase (GSR). The operation to delete water and add hydrogen atoms was accomplished by AutoDockTools software. Then, the molecular docking study was performed using the AutoDock Vina program. The coordinates of the target active pocket were listed in Supplementary Table S1. The docking results with the highest scores were visualized by PyMOL software.

## Results

### Pathological changes

**Figure 2:**
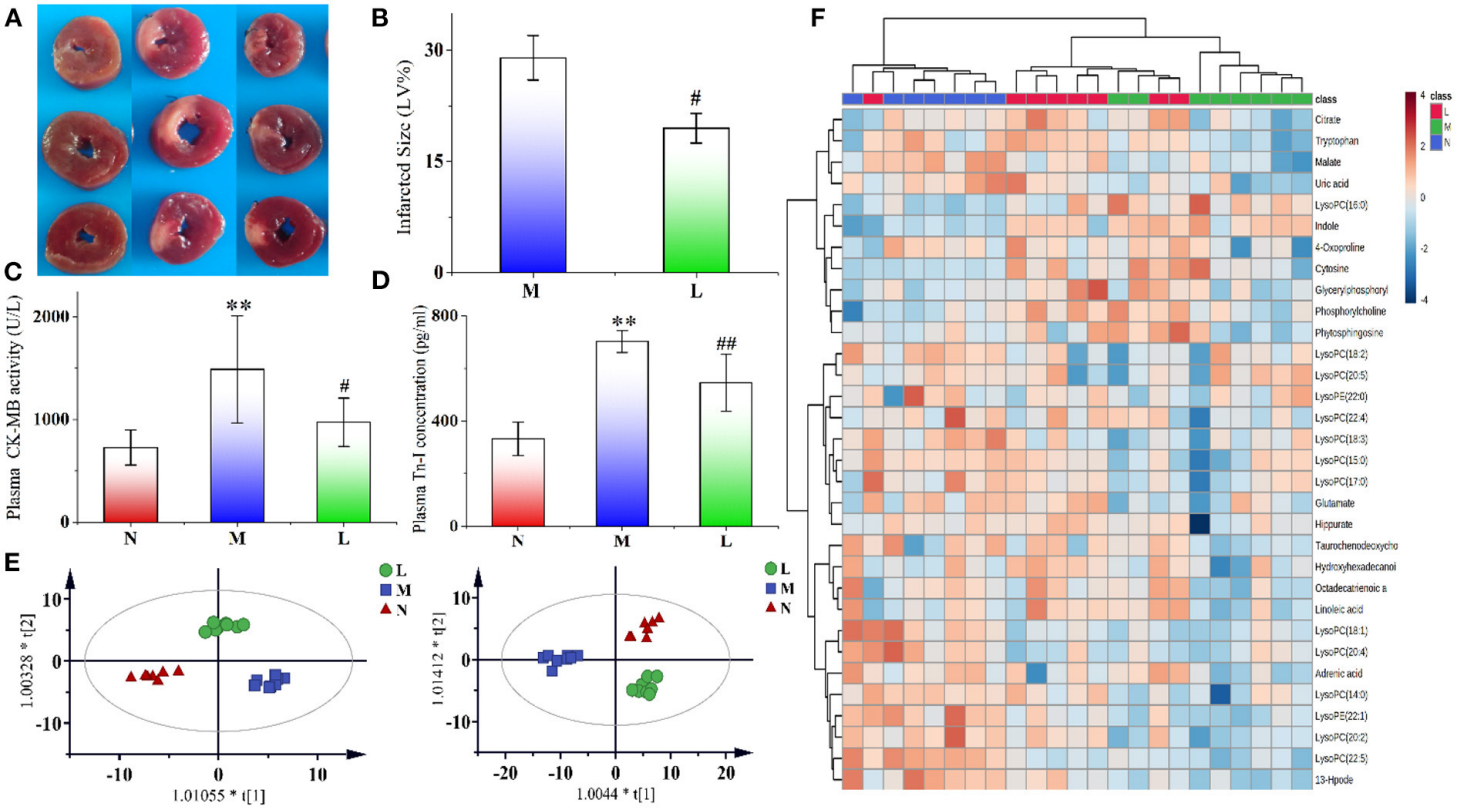
**(A)** Typical cross sections of rat myocardium in the N, M, and L groups. We selected three typical myocardial tissue samples from each group for presentation. Areas stained red indicated normal myocardium, while areas not stained red and that were pale were infarcted. **(B)** Effect of leonurine on myocardial infarction size in rats with AMI. **(C,D)** Effects of leonurine on plasma CK-MB and Tn-I levels in AMI rats. **(E)** OPLS-DA score plots of the N, M, and L groups (*n* = 8) from metabolomics in positive (*R*^2^ = 0.915, *Q*^2^ = 0.772) and negative (*R*^2^ = 0.938, *Q*^2^ = 0.755) modes. Red triangles, blue diamonds and green circles indicate the N, M, and L groups, respectively. **(F)** Hierarchical clustering heatmap showing the changes in potential biomarker content in the plasma of rats in the N, M, and L groups. The three groups achieved basic separation based on 32 potential biomarkers. The N and M groups were completely separated, and the L group approached N group. N, the sham-operated group; M, the acute myocardial ischemia model group; L, the leonurine administration group. **p < 0.01 *vs*. the N group; #*p* < 0.05 and ##*p* < 0.01 *vs*. the M group.

The cross sections of the typical infarct myocardial sections of rats in the three groups are shown in Figure 2A. Areas stained red indicated normal myocardium, while areas not stained red and that were pale were infarcted. The myocardial tissue of the N group rats was all stained red without infarction areas, while the myocardial infarction areas of the M group rats were obvious. After leonurine intervention, the area of myocardial infarction was significantly reduced. ImageJ software was used to analyze the size of myocardial infarction. The results showed that the administration of leonurine effectively improved MI in rats and reduced the size of myocardial infarction (*p-*value < 0.05). The results are shown in Figure 2B.

### Effects of leonurine on CK-MB and Tn-I

It has been widely accepted that CK-MB and TN-I are important indicators of myocardial ischemia (29–31). The effects of leonurine on plasma CK-MB activity and Tn-I concentration in the rats were shown in Figures 2C,D. Compared with the N group, the CK-MB activity and Tn-I concentration in the M group significantly increased (*p-*value < 0.01), indicating the successful establishment of the AMI model. However, the CK-MB activity and Tn-I concentration decreased significantly after leonurine intervention, indicating that leonurine could significantly improve the AMI in rats.

### Metabolomics analysis

After Pareto scaling and log transformation, unsupervised clustering PCA was used to evaluate the performance of different groups. In the score plots of PCA (Supplementary Figure S1A), the QC samples were distributed in the near center in both positive and negative modes. The samples of the M group were significantly separated from those of the N group, indicating that AMI caused significant changes in metabolite levels in rats. Compared with the M group, the L group approached the N group, indicating that the levels of some metabolites in AMI rats were reversed after intraperitoneal injection of leonurine. The *R*^2^ values in positive and negative modes were 0.470 and 0.561, respectively.

To explore the differential metabolites between groups, an OPLS-DA model was also established for analysis. In the score plots of OPLS-DA in Figure 2E and Supplementary Figures S2B,C, complete separation was achieved between the N and M groups, as well as between the L and M groups. The values of *R*^2^ and *Q*^2^ in the OPLS-DA model in both positive and negative modes were all >0.755. Permutation testing for the OPLS-DA model was also performed, and the results are shown in Supplementary Figure S2. All the candidate metabolites with a VIP value >1 were considered differential metabolites, and then they were identified by comparing their MS information with standard substances and available online biochemical databases. Eighteen metabolites were detected in positive ion mode, and 14 were detected in negative ion mode. Detailed information on the 32 potential biomarkers is listed in Supplementary Table S2. The HCA of 32 potential biomarkers is shown in Figure 2F, and related pathways with influence coefficient are shown in Supplementary Figure S3A. Pathways with an impact value >0.1 were considered to be closely related to AMI, and leonurine exhibited a protective effect on the disorder via the regulation of these pathways, including linoleic acid metabolism, glycerophospholipid metabolism, glutamate metabolism, tryptophan metabolism, and the TCA cycle. Among them, glycerophospholipid metabolism and tryptophan metabolism should receive attention, since they were detected in the plasma of both rat samples with AMI and clinical samples of patients with hyperlipidemia, as shown in Supplementary Figure S3B.

### Network pharmacology analysis

**Figure 3:**
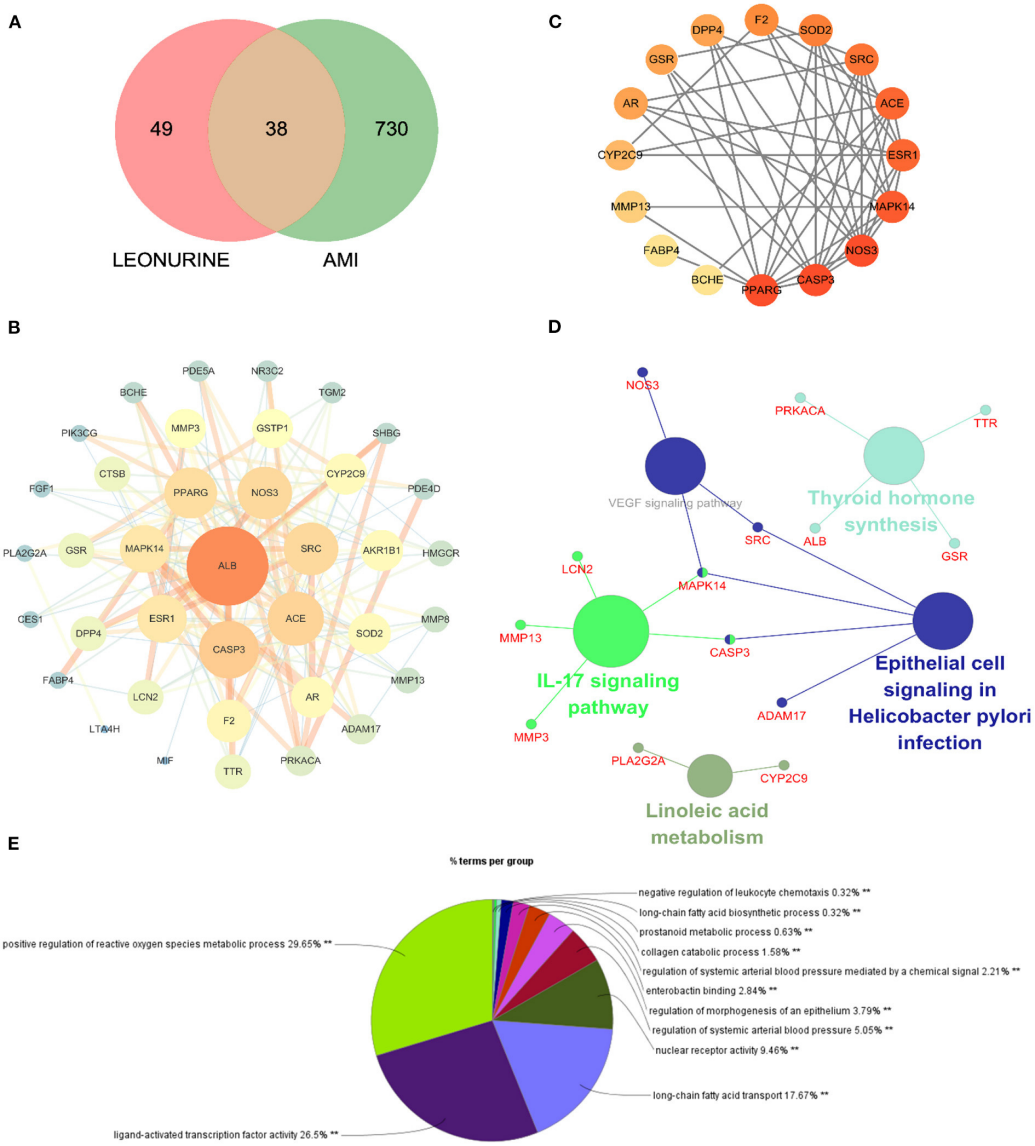
Network pharmacology of leonurine against AMI. **(A)** Venn diagram of the intersection targets between leonurine and AMI. **(B)** The PPI network of leonurine against AMI. **(C)** Analysis of the hub genes by the CytoHubba plugin. The darker the color of the nodes, the greater the degree value. **(D)** KEGG pathway enrichment analysis by ClueGO with a *p*-value < 0.05. **(E)** GO enrichment analysis of potential targets by ClueGO.

Network pharmacology was used to predict the potential targets of leonurine against AMI. A total of 87 targets of leonurine and 768 targets of AMI were obtained from the databases mentioned in Section 2.7 Network pharmacology research. Their shared targets were considered potential targets of leonurine against AMI (Figure 3A). Then, a PPI network was built by the SRING database to further analyze the complex mechanisms of leonurine against AMI. The obtained PPI data was visualized by Cytoscape software (Figure 3B). The CytoHubba plugin in Cytoscape software was used to calculate the hub genes. The top 20 genes were obtained in each algorithm in the CytoHubba plugin (Supplementary Figure S4). The overlapping genes in these algorithms were considered the hub genes (Figure 3C). A total of 16 hub genes (PPARG, CASP3, NOS3, MAPK14, ESR1, ACE, SRC, SOD2, F2, DPP4, GSR, AR, CYP2C9, MMP13, FABP4, BCHE) were thus obtained. The GO and KEGG pathway enrichment analyses were completed by the ClueGO plugin in Cytoscape software, and top terms such as positive regulation of reactive oxygen species metabolic process (GO:2000379), ligand-activated transcription factor activity (GO:0098531), long-chain fatty acid biosynthetic process (GO:0042759), nuclear receptor activity (GO:0042759), regulation of systemic arterial blood pressure (GO:0003073) and regulation of morphogenesis of an epithelium (GO:1905330) are shown in Figure 3D. According to the KEGG enrichment analysis, the pathways significantly affected were linoleic acid, IL-17 signaling pathway, thyroid hormone synthesis, and epithelial cell signaling in Helicobacter pylori infection.

### Integrated analysis of metabolomics and network pharmacology

**Figure 4:**
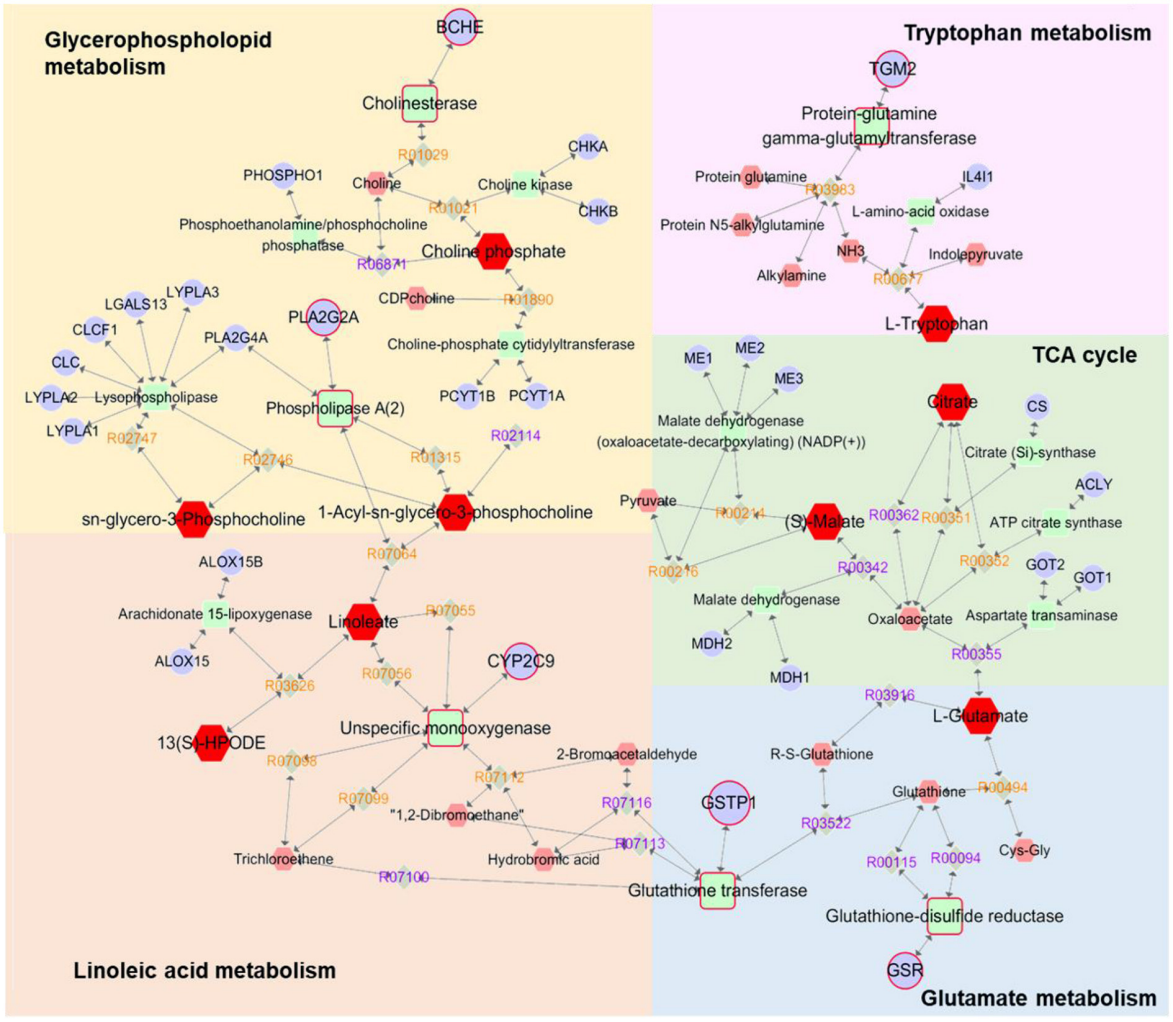
Compound-reaction-enzyme-gene networks of the key metabolites and targets. The red hexagons are differential metabolites, gray diamonds are reactions, green round rectangles are proteins and purple circles are genes. The key metabolites, proteins and genes were magnified.

A compound-reaction-enzyme-gene network (Figure 4) was constructed by performing an integrative analysis of the metabolomics-acquired differential metabolites and the hub genes obtained from network pharmacology using the Metscape plugin in Cytoscape software. The network intuitively showed the relationships between differential metabolites and their upstream hub genes. A total of six key targets (GSR, CYP2C9, BCHE, GSTP1, TGM2, and PLA2G2A) were identified in the network map, corresponding to seven differential metabolites (glycerylphosphorylcholine, lysophosphatidylcholine, choline phosphate, linoleic acid, 13-HpODE, tryptophan and glutamate), involved in four important metabolic pathways (glycerophospholopid metabolism, linoleic acid metabolism, tryptophan metabolism and glutamate metabolism). Among these genes, GSR, CYP2C9, and BCHE were hub genes screened by network pharmacology.

### Molecular docking

**Figure 5:**
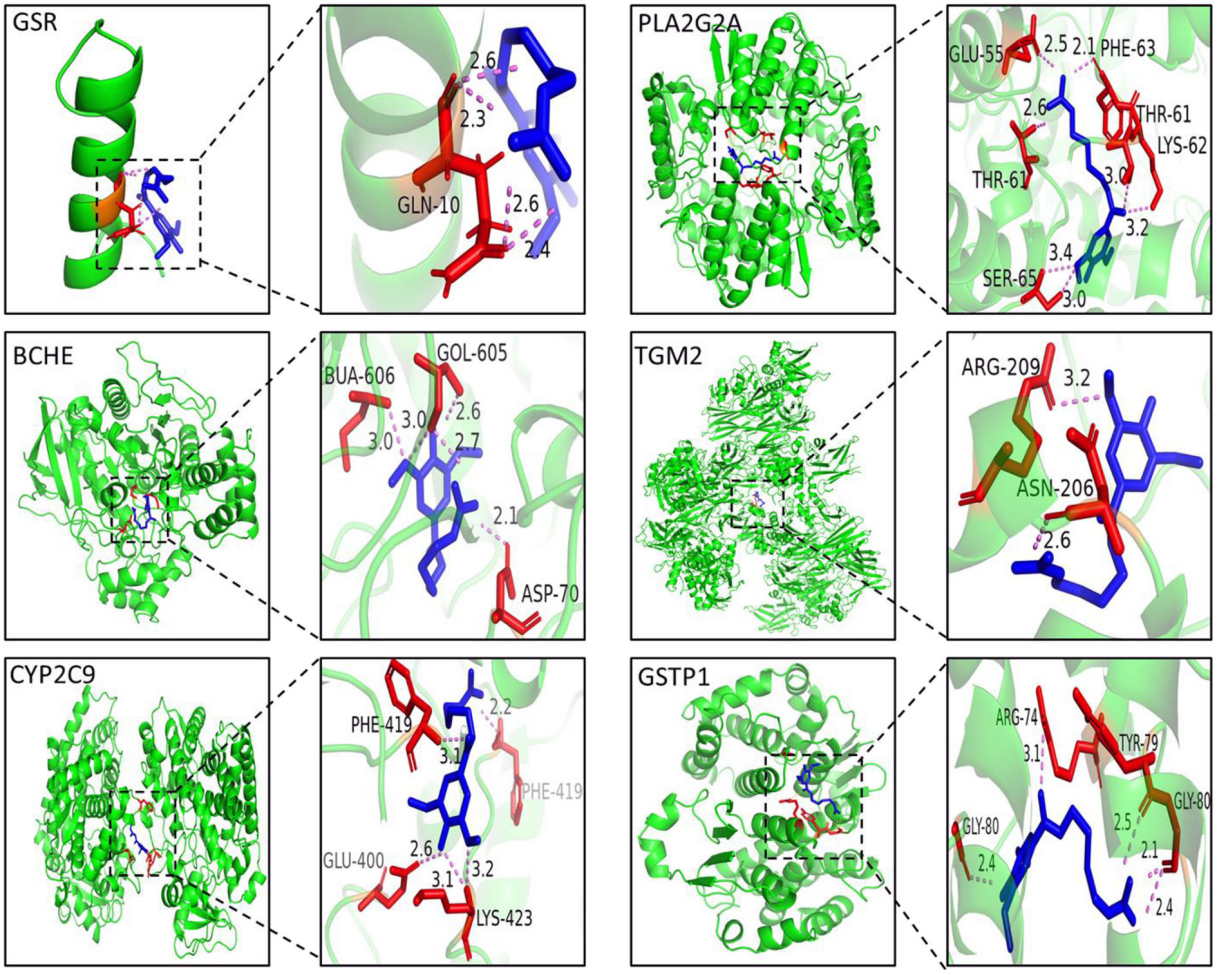
The 3D interaction diagrams of leonurine and GSR, BCHE, CYP2C9, PLA2G2A, TGM2 and GSTP1.

In this work, molecular docking technology was also used to predict the binding ability between leonurine and key targets (GSR, CYP2C9, and BCHE). The results of the docking experiments are shown in Figure 5. In detail, leonurine and the key targets were interacted with hydrogen-bonds, and the binding energies were −4.7 (GSR), −6.5 (BCHE) and −7.2 (CYP2C9) kcal/–mol. As shown in Figure 5A, leonurine made hydrogen-bonding interactions with GLN-10 of GSR at the active sites. To BCHE, leonurine made hydrogen-bonding interactions with ASP-70, GOL-605 and BUA-606 at the active sites. CYP2C9 interacted with leonurine by hydrogen bonding via the active site of four amino acid residues (PHE-419, LYS-423, GLU-400 and PHE-419). The docking results indicated a good affinity between leonurine and the targets.

## Discussion

In this study, an integrated network pharmacology and metabolomics strategy was used to explore potential targets and metabolic pathways of leonurine against AMI, and a molecular docking technique was used to validate the key targets. Leonurine is a promising chemical component with multiple effects. Our team's study on leonurine treatment for hyperlipidemia has now entered clinical phase III. According to our metabolomics study on patients with clinical hyperlipidemia (one of the important risk factors for CHD), leonurine showed a great influence on glycerophosphololipid metabolism and tryptophan metabolism (Supplementary Figure S3). The regulation of leonurine on these two metabolic pathways was also observed in this study, which greatly aroused our interest. In the metabolomics experiment, a reduced level of glycerylphosphorylcholine (GPC) was observed. GPC is a small molecule that is normally water-soluble *in vivo* that decomposes into choline and glycerol phospholipids under the action of related enzymes, in which choline participates in the biosynthesis of acetylcholine, and glycerol phospholipids are involved in phosphatidylcholine (PC) synthesis. PCs can be catalyzed by phospholipase A2 (PLA2G2A) and partially hydrolyzed into lysophosphatidycholines (LysoPCs) (32, 33). In the metabolomics study, a significant decrease in multiple LysoPC species was observed in the AMI group of rats, which was consistent with a previous report (34). However, the LPC levels significantly increased after leonurine intervention. Interestingly, PLA2G2A was predicted to a potential target for leonurine through networked pharmacology studies, whose activity is closely related to LPC levels. Therefore, a molecular docking technique was used to analyze the binding ability between leonurine and PLA2G2A. The results in Figure 5 show that leonurine interacted with PLA2G2A by hydrogen bonds at the active site, with binding energies of −7.4 kcal/–mol, indicating the potential of PLA2G2A to be a target of leonurine. Taken together, we speculated that leonurine may promote the hydrolysis of PCs into LPCs by acting on PLA2G2A to increase the levels of LPCs. Choline phosphate, another differential metabolite found in metabolomics study, was also involved in glycerophospholipid metabolism. A decrease in choline phosphate levels at AMI also interfered with the levels of LPCs. A network pharmacology study predicted that cholinesterase (BCHE) was one of the hub genes against AMI. Leonurine might regulate the levels of choline phosphate by acting on BCHE and thus affect the levels of LPCs. Therefore, leonurine may regulate the levels of LPCs by multiple targets.

Fatty acids (FAs) are the largest energy reservoir within the body. They can be oxidized in mitochondria by β-oxidation, producing acetyl-CoA, and are involved in the Krebs cycle (TCA) to provide energy. In AMI, aerobic respiration was limited, which suppressed the TCA cycle and interfered with the β-oxidation process of FAs, leading to the metabolic disorder of FAs and decreased levels of multiple FAs (35, 36). Decrease in unsaturated fatty acids such as linoleic acid, octadecatrienoic acid, hydroxyhexadecanoic acid, adrenic acid and 13-HpODE were observed in the metabolomics experiment. Among them, linoleic acid participates in the synthesis of LPCs, and the change in its level is correlated with the level of LPCs. Cytochrome P450 2C9 (CYP2C9) is a monooxidase that is involved in the metabolism of linoleic acid and generates epoxyoctadecanoic acids (EpOMEs). EpOMEs are further catalyzed by soluble hydroxyhydrolase (sEH) to produce dihydroxyoctadelactic acid (DiHOMEs). EpOMEs and DiHOMEs are closely related to the recovery of cardiac function after AMI (37). The network pharmacology results revealed that CYP2C9 (hub gene) was a possible target for leonurine. Therefore, it was possible that leonurine reduced the degradation of linoleic acid by inhibiting CYP2C9 activity, and thus reduced the harm to myocardial tissue during AMI.

Abnormal decreases in citrate and malate levels in rats with AMI were also observed in this work. As important metabolic intermediates in the TCA cycle, the reduced levels of citrate and malate indicated the inhibition of the TCA cycle. This might be related to the limitation of aerobic respiration during AMI in rats. Hypoxia in mitochondria and the reduction in substrate supply could cause disorders of the myocardial energy metabolism pathway, and the TCA cycle, as the core of energy metabolism, was obviously disturbed (38).

In fact, metabolic drugs improving the function of ischemic myocardium play an important role in the therapy for CHD. One of the representative metabolic drugs is trimetazidine, which could partial blockade of lipid β-oxidation and stimulate glucose oxidation (39). However, the antihypoxic effects of trimetazidine can be realized only in moderate hypoxia due to regulatory limitation of lipid utilization. Unlike trimetazidine, leonurine show obvious regulation on both TCA cycle and FAs metabolism. Khazanov et al. (40) found that the optimization of energy formation processes in mitochondria using natural mitochondrial metabolites, such as succinate and malate showed more effectively than antihypoxant trimetazidine prevented functional and metabolic disorders in rat myocardium during AMI. This encourages us that leonurine has a good future on the clinical application of AMI therapy.

Glutamate participates in the malate-aspartate shuttle and converts pyruvate to alanine rather than lactic acid; thus, glutamate might be the preferred myocardial fuel for AMI (41, 42). Furthermore, the reduced 4-oxoproline level in this experiment was also associated with an abnormal metabolism of glutamate. The results of a network pharmacology study showed that GSTP1 and GSR have the potential to be targets of leonurine in glutamate metabolism. The glutathione (GSH) redox system is an important antioxidant defense system in cardiomyocytes (43). The absence of GSH aggravated the injury of myocardial tissue, and the reduction in GSH concentration was associated with the decreased activity of glutathione reductase (GSR) (44). Leonurine may repair the myocardial damage caused by AMI by upregulating the concentration of GSH through the activation of GSR. Glutathione S-transferase Ps (GSTPs) protect cardiomyocytes by removing cytotoxic dehydes such as acrolein from the heart (49). Unlike other GSTPs, GSTP1 has the greatest cardio-selectivity and plays a protective role during AMI. Molecular docking results showed that the binding energy between leonurine and GSTP1 was −6.5 kcal/–mol (Figure 5), indicating that GTPS1 was a potential target for leonurine to prevent AMI.

The effect of leonurine on metabolic disorders of the tryptophan pathway was also observed in both a previous metabolomics study of patients with clinical hyperlipidemia and this work. As an antioxidant, tryptophan was reported to alleviate hypoxic myocardial damage (45). Clinical studies have shown that the tryptophan metabolism pathway is closely related to the onset and development of AMI. For example, Petras et al. (46) and Chacko et al. (47) observed a significant reduction in tryptophan levels in patients with AMI 5 min before onset and within 12 h after onset through a metabolomics study. A decrease in tryptophan levels was also observed in this metabolomics study. The network pharmacology study predicted protein-glutamine gamma-glutamyl transferase 2 (TGM2) as a possible target for leonurine in tryptophan metabolism. TGM2 is a member of the transglutaminase (TG) family that contributes to the stabilization or repair of the vasculature. Griffin et al. (48) observed more myocardial fibrosis in TGM2 knockout mice (ApoEe/TGM2/F13a1 knockout and Tgm2/F13a1 knockout mice) than in ApoEe/F13a1 double-knockout and F13a1 single-knockout mice. TGM2 could also promote ATP synthesis and thus limit damage after AMI (49). Thus, the binding ability between leonurine and TGM2 was analyzed by molecular docking. The docking result in Figure 5 shows that the interaction between TGM2 and leonurine was achieved by hydrogen bonding with a binding energy of −6.2 kcal/–mol. In conclusion, we predicted that the tryptophan pathway was greatly affected in rats with AMI, and leonurine probably repaired the myocardial injury caused by AMI by acting on TGM2.

**Figure 6:**
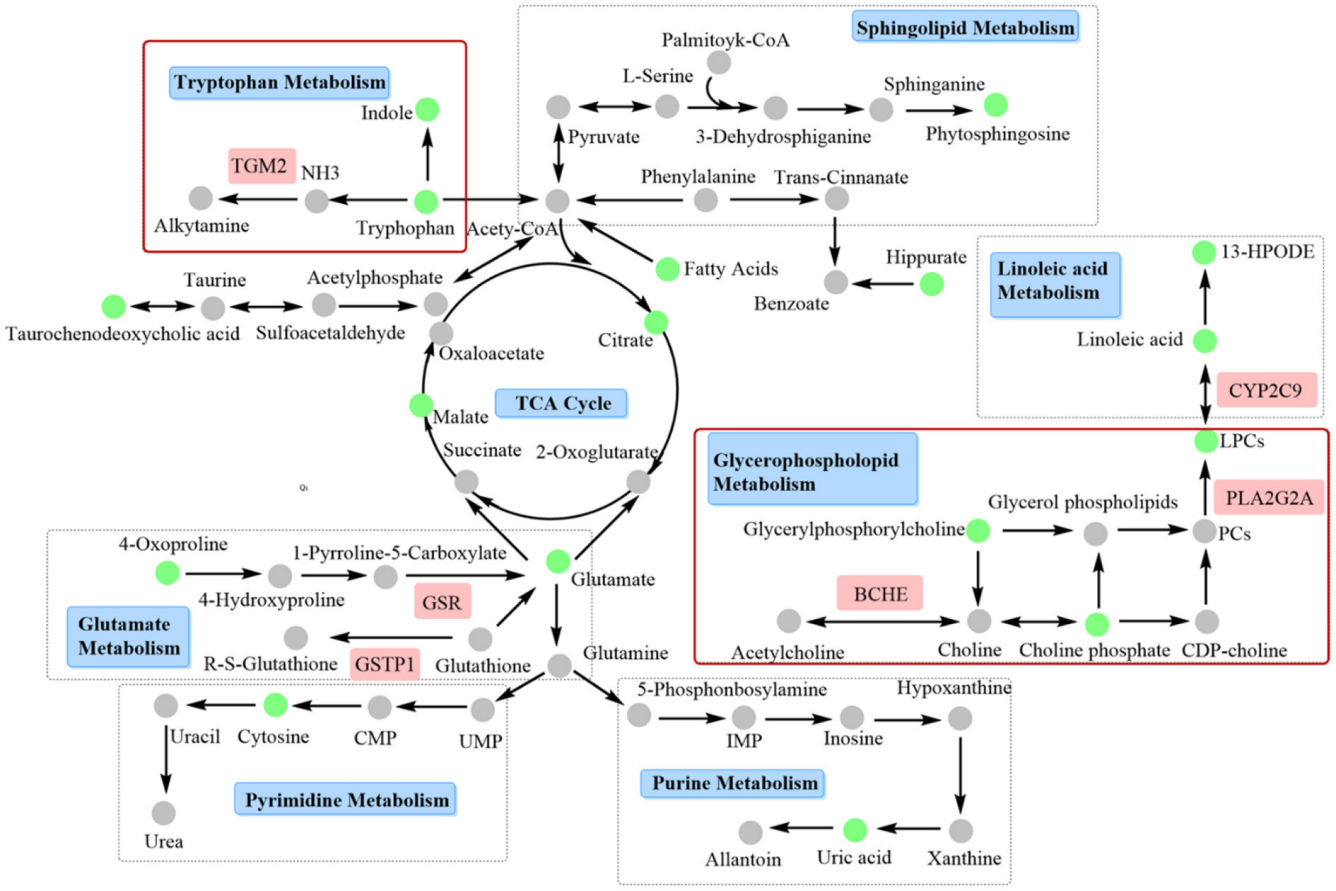
Interaction network based on metabolomics and network pharmacology. Tryptophan and glycerophospholipid metabolism, and related targets such as TGM2, BCHE, and PLA2G2A should be paid more attention, since these two pathways may be closely related to the clinical efficacy of leonurine against AMI.

Decreases in other differential metabolites, such as hippurate, taurochenodeoxycholic acid, phytosphingosine, uric acid and cytosine were also observed in metabolomics experiments. Hippurate is the metabolite of phenylalanine, and its decreased level indicates abnormalities in the phenylalanine metabolic pathway (38). Taurine was reported to have protective effects on cardiomyocytes by maintaining calcium (Ca^2+^) homeostasis, regulating osmotic homeostasis, and mediating antioxidant and anti-apoptotic activities (41, 42). In this study, a decrease in the level of taurochenodeoxycholic acid was observed, which, as a precursor to taurine production, might be one of the causes of abnormal taurine metabolism. Sphingolipid-mediated signaling pathways were also reported to have an important role in cardiovascular pathological physiology and were considered a potential target for MI injury (43). In this study, a decreased level of phytosphingosine was found in AMI rats, which was consistent with the experimental results of Qi et al. (44). After leonurine administration, the level of phytosphingosine recovered, indicating that leonurine also had a regulatory effect on sphingolipid metabolism. Myocardial energy disorders inevitably affect DNA and protein synthesis, resulting in purine and pyrimidine metabolism disorders. It was reported that the level of xanthosine decreased in AMI. As the final product of purine metabolism, uric acid is metabolized by xanthosine and xanthine. Thus, decreases in xanthosine and xanthine resulted in a decrease in the levels of uric acid (35). In addition, cytosine, an intermediate product of pyrimidine metabolism, decreased, indicating that pyrimidine metabolism was also affected by myocardial ischemia. Figure 6 illustrates the underlying mechanism of leonurine against AMI by integrating network pharmacological and metabolomic results.

We preliminarily predicted the potential signaling pathway of leonurine against AMI in this study. Although the experimental results of plasma metabolomics is a powerful support of our prediction of the potential targets, further validation of the predicted targets should be further verified to clarify the mechanism of leonurine against AMI. Especially, tryptophan and glycerophospholipid metabolism, and related targets such as TGM2, BCHE and PLA2G2A should be paid more attention, since the regulation of leonurine on these two pathways was also observed in our previous metabolomics study of clinical hyperlipidemia patients. These two pathways may be closely related to the clinical efficacy of leonurine against AMI.

## Conclusion

In this study, we used an integrated metabolomics and network pharmacology strategy to explore the targets and related pathways of leonurine against AMI. A total of four major metabolic pathways were found, namely, glycerophospholipid metabolism, linoleic acid metabolism, tryptophan metabolism and glutamate metabolism, and six possible targets were filtered, three of which were hub genes in network pharmacology. Among them, tryptophan and glycerophospholipid metabolism should be given more attention, because disturbances of these two metabolic pathways were also found in clinical patients with hyperlipidemia. Hyperlipidemia is one of the main risk factors for CHD, and leonurine showed significant regulatory effects on tryptophan and glycerophospholipid metabolism disorders in both AMI rats and clinical hyperlipidemia patients. This work provides a valuable basis for the research and development of leonurine as an anti-AMI drug.

## Data availability statement

The original contributions presented in the study are included in the article/Supplementary material, further inquiries can be directed to the corresponding author/s.

## Ethics statement

The animal study was reviewed and approved by the Institutional Animal Care and Use Committee of Nantong University.

## Author contributions

QingZ conceived and designed the project. YZ provided leonurine sample and consultation. WR and QinbZ performed network pharmacology experiment. WR and JL did the main work. WR integrated the data and wrote the manuscript. JL, XQ, and XZ performed metabolomics experiment. LW performed animal experiments. SL analyzed histological samples. TL guided data processing. All authors had approved the manuscript.

## Funding

This research was funded by grants from National Natural Science Foundation of China (82071238, 81971243, and 81973320), Natural Science Foundation of Jiangsu Province (BK20181459), and the Macau Science and Technology Development Fund (0067/2018/A2, 033/2017/AMJ, 0007/2019/AKP, 0052/2020/A, and 0011/2020/A1). Large Instrument and Equipment Fund of Nantong University (KFJN2267).

## Conflict of interest

The authors declare that the research was conducted in the absence of any commercial or financial relationships that could be construed as a potential conflict of interest.

## Publisher's note

All claims expressed in this article are solely those of the authors and do not necessarily represent those of their affiliated organizations, or those of the publisher, the editors and the reviewers. Any product that may be evaluated in this article, or claim that may be made by its manufacturer, is not guaranteed or endorsed by the publisher.
