# Supplementary material for: Investigation of the protective mechanism of leonurine against acute myocardial ischemia by an integrated metabolomics and network pharmacology strategy
Source: Front Cardiovasc Med. 2022 Aug 22;9:969553. doi: 10.3389/fcvm.2022.969553 (PMC9441747; doi:10.3389/fcvm.2022.969553)
Supplement: Supplementary file 1 [file Data_Sheet_1.PDF]

Supplementary Material

1 Supplementary Figures and Tables

1.1 Supplementary Figures

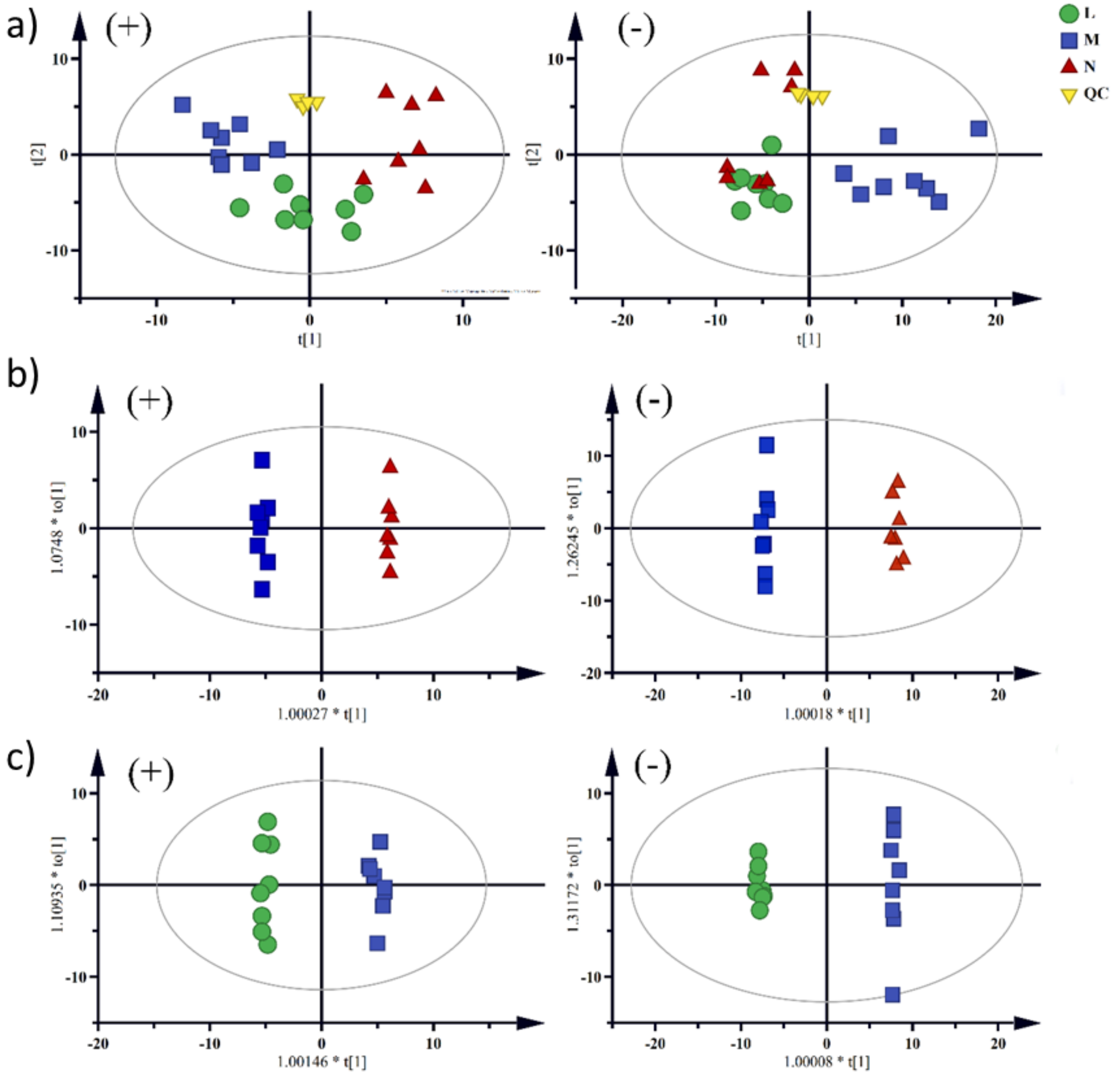

Supplementary Figure 1. Score plots of metabolomics of the plasma samples. a): The PCA score plots of QC, N, M and L groups in positive (R^2=0.470) and negative (R^2=0.561) modes. b): The OPLS-DA score plots of N and M groups in positive (R^2=0.998, Q^2=0.868) and negative (R^2=0.998, Q^2=0.874) modes. c): The OPLS-DA score plots of M and L groups in positive (R^2=0.992, Q^2=0.774) and negative (R^2=0.999, Q^2=0.868) modes. n=8, Yellow inverted triangle, red triangle, blue diamond and green circle were labelled as QC, N, M and L group, respectively. QC: quality control samples; N: the sham-operated group; M: the acute myocardial ischemia model group; L: the leonurine administration group.

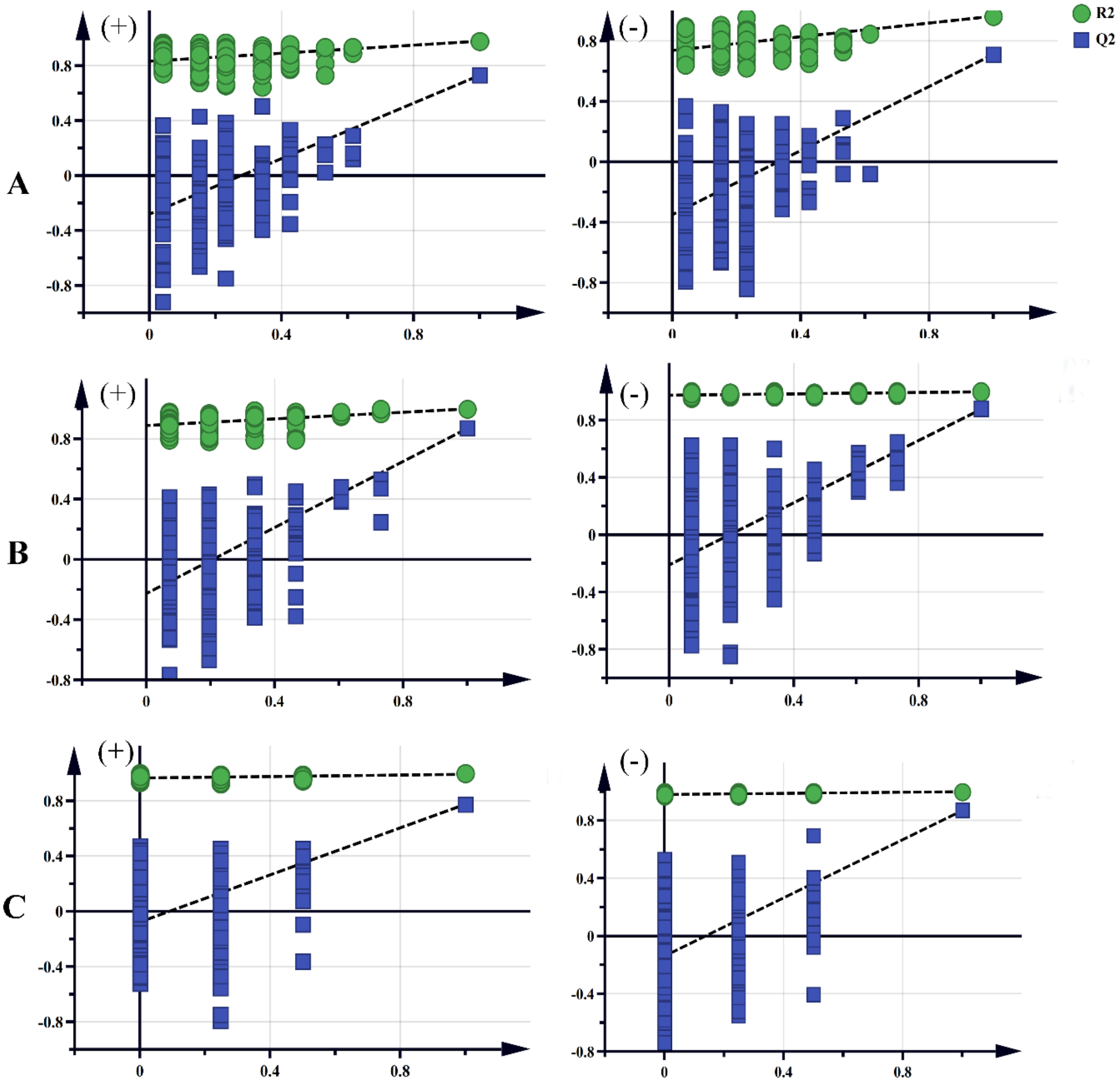

Supplementary Figure 2. Verification plots of 200 permutation tests for OPLS-DA. A: Plots for N, M and L groups in positive (intercepts R^2=0.831, Q^2=–0.238) and negative(intercepts R^2=0.737, Q^2=–0.35) modes; B: Plots for N and M groups in positive (intercepts R^2=0.888, Q^2=–0.288) and negative (intercepts R^2=0.974, Q^2=–0.211) modes; C: Plots for M and L groups in positive (intercepts R^2=0.965, Q^2=−0.0781) and negative (intercepts R^2=0.980, Q^2=–0.14) modes. N: the sham-operated group; M: the acute myocardial ischemia model group; L: the Leo administration group. Green and blue dots stand for R^2 and Q^2, respectively.

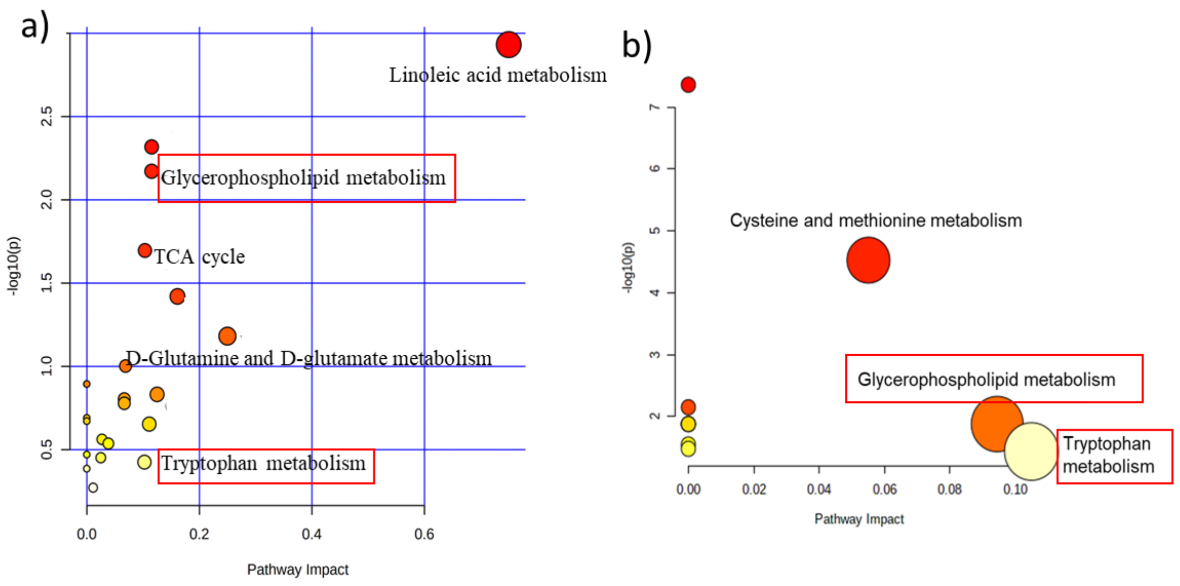

Supplementary Figure 3. An overview of pathway analysis in plasma of rat samples with AMI (a) and clinical samples of patients with hyperlipidemia (b) after the leonurine intervention. Glycerophospholipid metabolism and tryptophan metabolism should receive attention, since they were detected in both human and rat plasma.

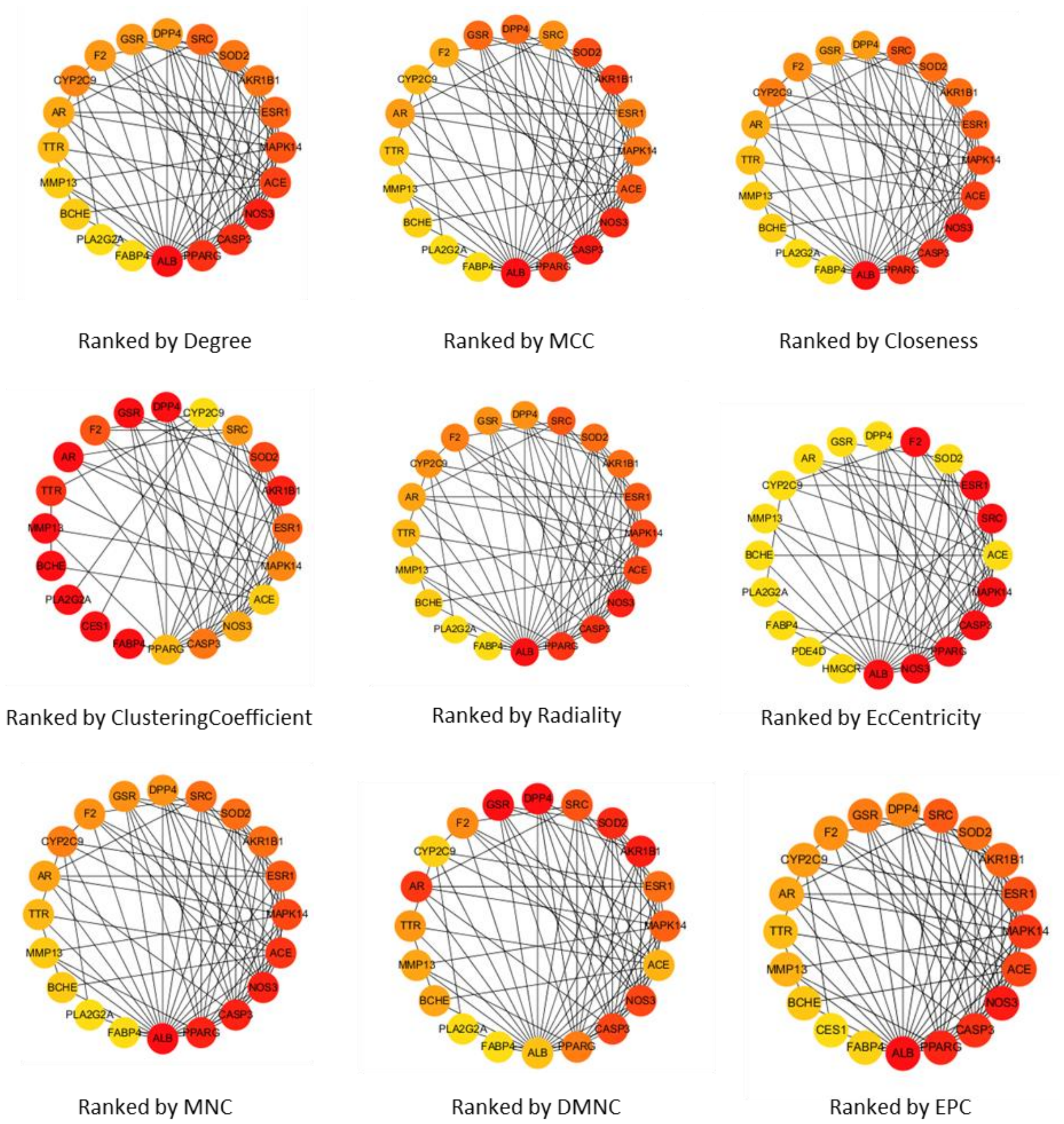

Supplementary Figure 4. Analysis of the hub genes (top 20) by CytoHubba plugin in Cytoscape software. The darker the color of the nodes, the greater the degree value.

1.2 Supplementary Tables

Supplementary Table 1. Parameters of molecular docking between leonurine and six targets.

Targets	center_x	center_y	center_z	size_x	size_y	size_z
GSR	83.149	40.487	26.887	18.1	14.1	27.2
BCHE	137.359	122.226	39.869	52.0	58.0	70.0
CYP2C9	37.19	61.368	28.334	104.0	74.0	76.0
GSTP1	19.744	12.057	16.302	56.0	68.0	60.0
PLA2G2A	-16.387	42.897	65.089	73.2	68.4	75.6
TGM2	10.351	116.596	216.938	126.0	126.0	126.0

Supplementary Table 2 The detailed information of the 34 potential biomarkers detected in positive and negative ion mode.

**No.**	**t_R(min)**	**MS(*m/z*)**	**ESI mode**	**Identification**	**Formula**	**VIP (N to M)**	**VIP (M to L)**	**FC (N to M)**	**FC (L to M)**	**Pathway**
1	0.78	191.01880	–	Citrate	$C_6H_8O_7$	1.04	1.16	1.59**	1.72##	TCA cycle
2	0.80	133.01402	–	Malate	$C_4H_6O_5$	1.72	1.17	3.69**	2.08#	TCA cycle
3$	0.75	146.04520	–	Glutamate	$C_5H_9NO_4$	1.12	1.18	1.91*	1.91#	Amino acid meta bolism
4	1.33	130.05028	+	4-Oxoproline	$C_5H_7NO_3$	1.36	1.26	1.56**	1.41#	Amino acid meta bolism
5$	4.52	203.08278	–	Tryptophan	$C_{11}H_{12}N_2O_2$	1.09	1.22	1.72**	1.74##	Amino acid meta bolism
6	4.52	116.0502	–	Indole	C_8H_7N	1.21	1.18	1.84**	1.74##	Amino acid meta bolism
7	5.16	178.05066	–	Hippurate	$C_9H_9NO_3$	1.36	1.44	1.90**	1.89##	Amino acid meta bolism
8	1.16	112.05008	+	Cytosine	$C_4H_5N_3O$	0.85	0.99	1.21**	1.25##	Pyrimidine Metabolism
9	1.19	167.02148	–	Uric acid	$C_5H_4N_4O_3$	1.56	1.18	2.68**	1.94#	Purine metabolism
10	7.61	498.29114	–	Taurochenodeoxych olic acid	$C_{26}H_{45}NO_6S$	1.34	1.18	3.03**	2.14##	Taurine metabolism
11$	8.96	318.30167	+	Phytosphingosine	$C_{18}H_{39}NO_3$	0.99	1.19	1.34*	1.49#	Sphingolipids metabolism
12	1.32	258.10889	+	Glycerylphosphoryl choline	$C_8H_{20}NO_6P$	0.97	1.29	1.31**	1.48##	Glycerophosphol opid metabolism
13	10.05	468.30869	+	LysoPC(14:0)	$C_{22}H_{46}NO_7P$	1.62	1.39	1.75**	1.54##	Glycerophosphol opid metabolism

14	10.20	518.32452	+	LysoPC(18:3)	$C_{26}H_{48}NO_7P$	1.57	1.25	1.77**	1.39##	Glycerophospholopid metabolism
15	10.58	482.32640	+	LysoPC(15:0)	$C_{23}H_{48}NO_7P$	1.61	1.51	1.79**	1.58#	Glycerophospholopid metabolism
16	10.75	520.34106	+	LysoPC(18:2)	$C_{26}H_{50}NO_7P$	1.39	0.99	1.50**	1.21#	Glycerophospholopid metabolism
17	10.75	542.32440	+	LysoPC(20:5)	$C_{28}H_{48}NO_7P$	1.23	0.94	1.38**	1.18#	Glycerophospholopid metabolism
18	11.03	570.35608	+	LysoPC(22:5)	$C_{30}H_{52}NO_7P$	2.07	1.21	2.42**	1.36#	Glycerophospholopid metabolism
19	11.25	522.35706	+	LysoPC(18:1)	$C_{26}H_{52}NO_7P$	1.50	1.06	1.59**	1.26#	Glycerophospholopid metabolism
20	11.45	544.33789	+	LysoPC(20:4)	$C_{28}H_{50}NO_7P$	1.47	0.96	1.55**	1.21#	Glycerophospholopid metabolism
21$	11.69	494.3269	–	LysoPC(16:0)	$C_{24}H_{50}NSO_7P$	1.34	1.18	3.03**	2.14##	Glycerophospholopid metabolism
22	11.72	510.35715	+	LysoPC(17:0)	$C_{25}H_{52}NO_7P$	1.37	1.39	1.58**	1.47##	Glycerophospholopid metabolism
23	11.76	548.37183	+	LysoPC(20:2)	$C_{28}H_{54}NO_7P$	2.19	1.99	2.84**	2.04##	Glycerophospholopid metabolism
24	11.97	536.37164	+	LysoPE(22:1)	$C_{27}H_{56}NO_7P$	2.23	1.64	2.93**	1.85##	Glycerophospholopid metabolism
25	12.06	184.07449	+	Choline phosphate	$C_5H_{15}NO_4P$	0.88	1.08	1.24*	1.29##	Glycerophospholopid metabolism
26	12.54	572.36920	+	LysoPC(22:4)	$C_{30}H_{54}NO_7P$	1.39	1.11	1.64**	1.34#	Glycerophospholopid metabolism
27	12.93	538.38580	+	LysoPE(22:0)	$C_{27}H_{56}NO_7P$	1.15	0.94	1.40**	1.23#	Glycerophospholopid metabolism
28	11.06	311.22186	–	13-Hpode	$C_{18}H_{32}O_4$	2.18	1.57	6.07**	2.53##	Linoleic acid metabolism
29	13.01	271.22797	–	Hydroxyhexadecanoic acid	$C_{16}H_{32}O_3$	1.06	1.18	1.70**	1.73##	Fatty acids metabolism

30	13.02	277.21640	–	Octadecatrienoic acid	$C_{18}H_{30}O_2$	1.48	1.16	2.54**	2.10#	Linoleic acid metabolism
31	13.60	279.23291	–	Linoleic acid	$C_{18}H_{32}O_2$	1.14	1.16	1.87*	1.94#	Linoleic acid metabolism
32	14.09	331.26431	–	Adrenic acid	$C_{22}H_{36}O_2$	1.67	1.80	3.65*	3.23##	Linoleic acid metabolism

N to M: *$p < 0.05$ and **$p < 0.01$; L to M: #$p < 0.05$ and ##$p < 0.01$; FC: fold change.

$：The compound was compared with-identified by standards.
